# Supplementary material for: Potential Effects of Indole-3-Lactic Acid, a Metabolite of Human Bifidobacteria, on NGF-Induced Neurite Outgrowth in PC12 Cells
Source: Microorganisms. 2020 Mar 12;8(3):398. doi: 10.3390/microorganisms8030398 (PMC7143819; doi:10.3390/microorganisms8030398)
Supplement: Supplementary file 1 [file microorganisms-08-00398-s001.zip › R1_Figure S1.pdf]

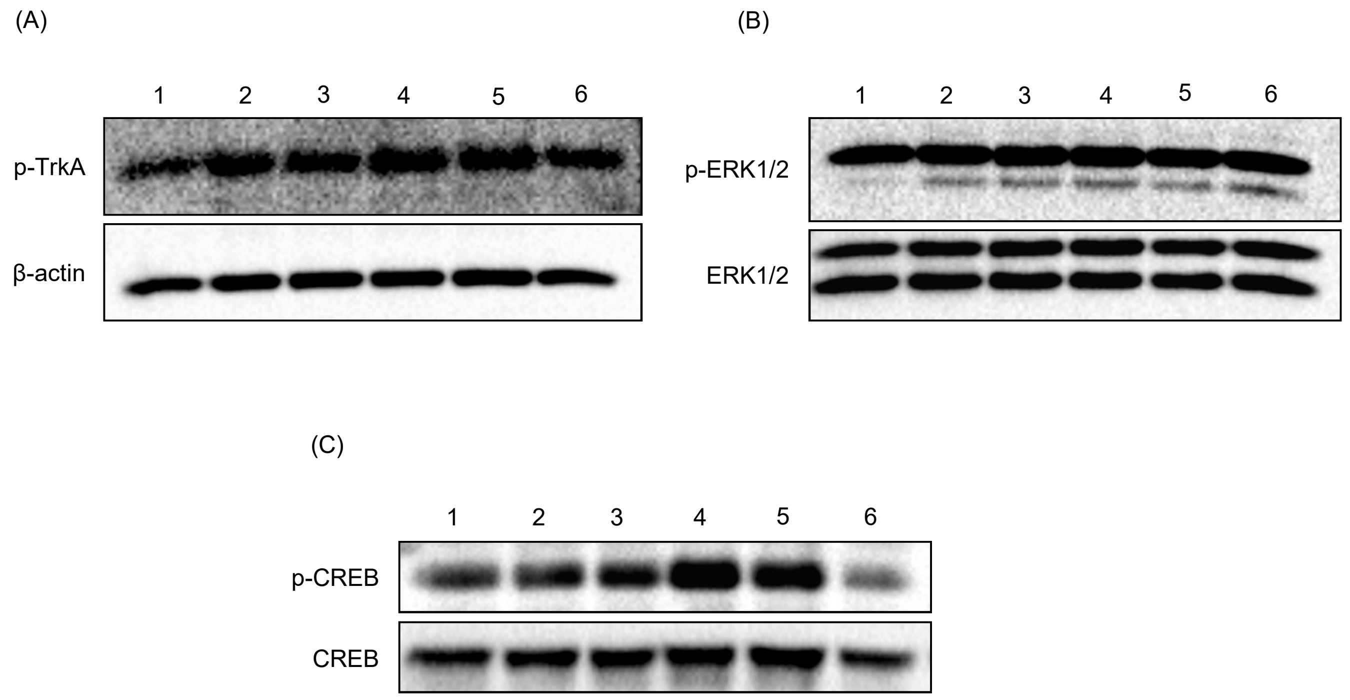

Figure S1. Western blot images of PC12 cells treated for 24 h with NGF (25 ng/mL) and ILA, IPA, or Trp (100 nM). The images show the phosphorylation of (A) TrkA, (B) ERK1/2, and (C) CREB in PC12 cells. Lane 1: non-treated control (NTC); Lane 2: Nerve growth factor (NGF)-treated control; Lane 3: Cells treated with NGF and papaverine hydrochloride as reference control (RC); Lane 4: Cells treated with NGF and indole-3-lactic acid (ILA); Lane 5: Cells treated with substance A; Lane 6: Cells treated with tryptophan (Trp).
