# Supplementary material for: Potential Effects of Indole-3-Lactic Acid, a Metabolite of Human Bifidobacteria, on NGF-Induced Neurite Outgrowth in PC12 Cells
Source: Microorganisms. 2020 Mar 12;8(3):398. doi: 10.3390/microorganisms8030398 (PMC7143819; doi:10.3390/microorganisms8030398)
Supplement: Supplementary file 1 [file microorganisms-08-00398-s001.zip › R1_Figure S2.pdf]

(A)

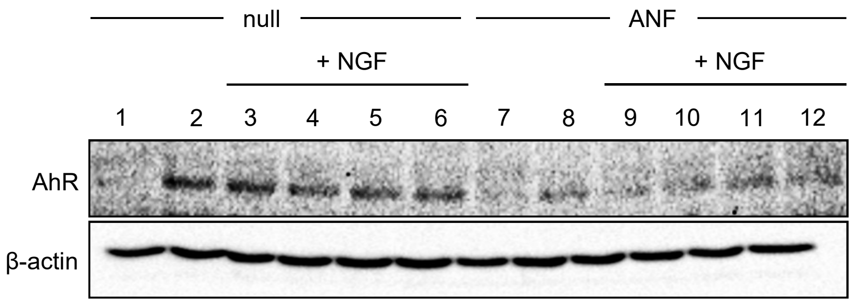

(B)

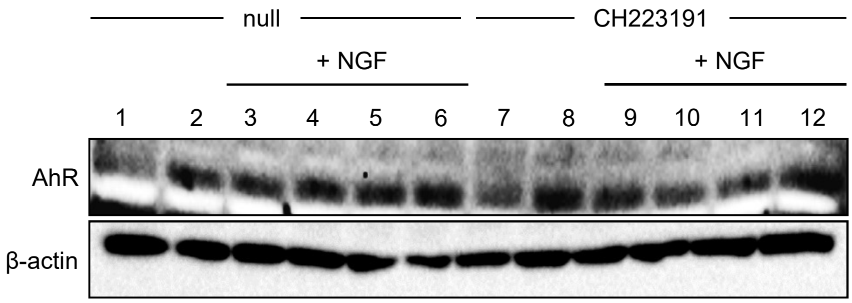

Figure S2. Western blot images of PC12 cells pre-treated with the AhR antagonists, (A) α-naphthoflavone (ANF; 1 μM) and (B) CH223191 (1 μM), for 1 h and treated for five consecutive days with NGF (25 ng/mL) and ILA, IPA, or Trp (100 nM). Non-pre-treated PC12 cells served as a null control. AhR protein (95 kDa) in PC12 cells was detected by Western blot analysis using a monoclonal antibody specific for AhR. The corresponding β-actin blot served as a loading control. Lane 1 & 7: non-treated control (NTC); Lane 2 & 8: Nerve growth factor (NGF)-treated control; Lane 3 & 9: Cells treated with NGF and papaverine hydrochloride as reference control (RC); Lane 4 & 10: Cells treated with NGF and indole-3-lactic acid (ILA); Lane 5 & 11: Cells treated with substance A; Lane 6 & 12: Cells treated with tryptophan (Trp).
